# EXAMINING THE IMPACT OF HEART DISEASE AND DIABETES ON THE MENTAL HEALTH OF OLDER MINORITY ADULTS

**DOI:** 10.1093/geroni/igac059.2832

**Published:** 2022-12-20

**Authors:** Sree Saroj, Sainath Panchagnula

**Affiliations:** Jackson State University, Jackson, Mississippi, United States; Jackson State University, Jackson, Mississippi, United States

## Abstract

As the world’s population ages and healthcare costs associated with the care of the elderly rise, it is pertinent to address these concerns. This study examines how older adults’ mental health is affected by chronic medical illnesses. It is hypothesized that minority older adults, on average, have worse mental and physical health outcomes than their majority counterparts. A secondary data analysis of the National Health and Aging Trends Study, revealed that older minority adults experience higher rates of depression than non-minority older adults, as measured by the Patient Health Questionnaire – 2 (PHQ-2). European-Americans significantly differ from African Americans (AA) and Hispanics regarding their level of anxiety, as measured by the Generalized Anxiety Disorder – 2 (GAD-2) measure. AA and Hispanics are significantly more likely to exhibit anxiety than their European-American counterparts. Minority older adults with a higher rate of surgeries are not substantially more likely to be depressed, according to the PHQ-2. Minority older adults who reported having a difficult time falling asleep are considerably more depressed than older minority adults who did not endorse depressive symptomatology on the PHQ-2. Minority older adults who had two or more social supports in their lives have lower levels of anxiety compared to older minority adults who have less than two social supports in their lives. The findings from this study serves as groundwork to promote equity between majority and minority older adults, which will improve their abilities to perform independent activities of daily living successfully.

